# Lobular breast cancer: molecular basis, mouse and cellular models

**DOI:** 10.1186/s13058-015-0517-z

**Published:** 2015-02-08

**Authors:** Matthias Christgen, Patrick WB Derksen

**Affiliations:** Institute of Pathology, Hannover Medical School, Carl-Neuberg-Straße 1, 30625 Hannover, Germany; Department of Pathology, Utrecht University Medical Center, Heidelberglaan 100, 3584 Utrecht, The Netherlands

## Abstract

Infiltrating lobular breast cancer (ILC) is the most common special breast cancer subtype. With mutational or epigenetic inactivation of the cell adhesion molecule E-cadherin (*CDH1*) being confined almost exclusively to ILC, this tumor entity stands out from all other types of breast cancers. The molecular basis of ILC is linked to loss of E-cadherin, as evidenced by human *CDH1* germline mutations and conditional knockout mouse models. A better understanding of ILC beyond the level of descriptive studies depends on physiologically relevant and functional tools. This review provides a detailed overview on ILC models, including well-characterized cell lines, xenograft tumors and genetically engineered mouse models. We consider advantages and limitations of these models and evaluate their representativeness for human ILC. The still incompletely defined mechanisms by which loss of E-cadherin drives malignant transformation are discussed based on recent findings in these models. Moreover, candidate genes and signaling pathways potentially involved in ILC development and progression as well as anticancer drug and endocrine resistance are highlighted.

## Introduction

**Figure 1:**
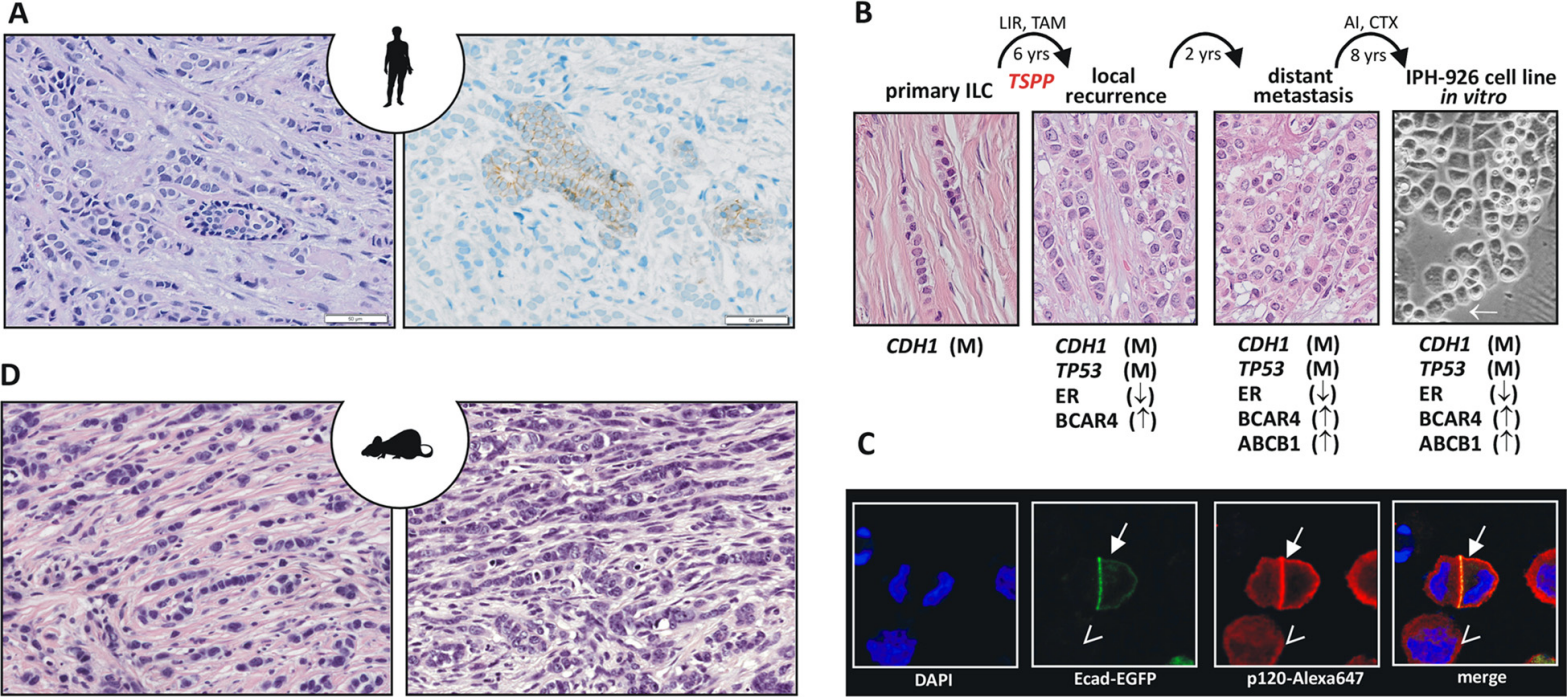
**Infiltrating lobular breast cancer, a human infiltrating lobular breast cancer cell line and a genetically engineered mouse model for infiltrating lobular breast cancer. (A)** Representative photomicrographs of infiltrating lobular breast cancer (ILC) stained with hematoxylin and eosin (left) or subjected to immunohistochemistry for E-cadherin (right). Note the E-cadherin-positive normal mammary gland duct surrounded by E-cadherin-negative ILC cells. **(B)** Molecular evolution of the IPH-926 ILC cell line. Photomicrographs show the histomorphology of the corresponding clinical tumor specimens and the IPH-926 cell line *in vitro.* Arrow highlights a single file linear cord reminiscent of primary ILC. AI, aromatase inhibitor; CTX, various conventional chemotherapies; LIR, local irradiation; TAM, tamoxifen; TSPP, transition to a secondary pleomorphic phenotype; yrs, years; M, mutational inactivation; ↑, overexpression; ↓, loss of expression. **(C)** Reconstitution of E-cadherin in ILC cells induces relocation of p120-catenin (p120) to the cell membrane. Shown are fluorescence images of IPH-926 cells transiently transfected with an E-cadherin–enhanced green fluorescent protein (Ecad-EGFP) expression construct and stained with an anti-p120-Alexa647 antibody. Closed arrow, cells with ectopic expression of Ecad-EGFP; open arrow, a cell without Ecad-EGFP. Note the accentuated membranous p120 staining in cells expressing Ecad-EGFP. DAPI, 4′,6-diamidino-2-phenylindole. **(D)** Mouse ILC from genetically engineered mouse models. Left, a tumor reminiscent of classic ILC; right, a pleomorphic mouse ILC. Both micrographs generated from the *WAPcre;Cdh1* ^*F/F*^ *;Trp53* ^*F/F*^ mouse ILC model.

Infiltrating lobular breast cancer (ILC) is the most common special breast cancer (BC) subtype and accounts for 10 to 15% of all mammary carcinomas. ILCs are defined by histomorphological characteristics, such as small, discohesive and nonpolarized tumor cells with little nuclear atypia and a single-file invasion pattern (Figure 1A). Although first termed ILCs in the 1940s [1], these tumors had been recognized as a histologically distinct entity (scirrhous spheroidal cell carcinoma) long before the terminus ILC became established [2].

In the 1980s, uvomorulin (E-cadherin) was discovered as a transmembrane glycoprotein that mediates cis and trans calcium-dependent homotypic cell adhesion in epithelial tissues, thereby controlling cell polarity and proper blastocyst formation during embryogenesis [3]. Together with the associated catenins, E-cadherin forms the adherens junction (AJ) on the apical side of the cell, where it links the plasma membrane to the actin and microtubule cytoskeleton [4]. E-cadherin is of pivotal relevance for two special tumor entities, namely ILC and diffuse gastric cancer (DGC). In the 1990s, it was reported that nearly all ILCs and their adjacent intraepithelial precursor lesions, termed lobular carcinoma *in situ* (LCIS), lacked E-cadherin expression [5,6]. E-cadherin is encoded by the *CDH1* gene on chromosome 16q22. Loss of E-cadherin in LCIS and ILC is due to somatic *CDH1* frameshift mutation and loss of heterozygosity or aberrant *CDH1* promoter methylation [7-9]. *CDH1* germline mutations are associated with the hereditary DGC syndrome [10] and ILC belongs to the tumor spectrum in these patients [11]. Cases of LCIS and ILC associated with *CDH1* germline mutation without gastric cancer are also increasingly recognized [12]. In addition to genetically engineered mouse (GEM) models (see below), these findings from medical genetics have provided evidence that E-cadherin functions as a tumor suppressor and that its inactivation underpins ILC etiology.

The molecular impact of E-cadherin inactivation has been studied extensively in the context of a biological process referred to as the epithelial-to-mesenchymal transition. While these studies have provided vast insight into the epigenetic mechanisms that can silence E-cadherin and their functional consequences, they also indicated that E-cadherin inactivation through transcriptional repressors is not the main driver of ILC development. Based on immunohistochemical and genetic studies, it is now established that loss of E-cadherin triggers secondary changes affecting several cadherin/catenin complex molecules. These changes include loss of β-catenin and aberrant cytoplasmic and/or nuclear localization of p120-catenin (p120) [13,14].

Almost all ILCs are estrogen receptor (ER)-positive and belong to the luminal or normal-like molecular subtype [15]. Their proliferation is slow and mostly estrogen dependent [16]. Overexpression or amplification of the *ERBB2* oncogene is rare, although somatic activating mutations have been reported [17]. In contrast, mutational activation of the *PIK3CA* oncogene is a dominant feature in ILC [18]. *TP53* mutations are rare [15], except for a more aggressive ILC variant termed pleomorphic ILC, which is more often ER-negative and occasionally ERBB2-positive [19,20]. Based on these findings and various genomic profiling studies [15,21,22], it became evident that ILC represents a biologically distinct entity.

A better understanding of ILC beyond the descriptive level of genetic and histopathological studies depends on clinically relevant models. This review provides an overview of mouse and human ILC models and their relevance for understanding ILC biology.

## Review

### Human ILC cell lines are a rare resource

Human BC cell lines are a powerful experimental tool. In many instances, information derived from *in vitro* models with BC cell lines has improved the understanding of cancer [23]. In other studies, potentially misleading data have been generated because cell lines were not representative for the tumor type investigated. Numerous studies have aimed to (re)classify BC cell lines in terms of their tumor origin and molecular properties. This revealed a lack of ILC cell lines [23,24]. Among more than 100 BC cell lines established to date, only seven can be tracked back to histologically confirmed or suspected primary ILCs (Table 1) [25-31]. Importantly, an ILC origin cannot be concluded simply based on the lack of E-cadherin expression. This is because *in vitro* culturing can induce epithelial-to-mesenchymal transition and a subsequent epigenetic silencing of E-cadherin [32]. In particular, this applies to BC cell lines with a basal molecular subtype that have most probably undergone epithelial-to-mesenchymal transition in culture. A renowned example is MDA-MB-231. The MDA-MB-231 cell line lacks E-cadherin due to hypermethylation, but is hardly comparable with ILC given its fast proliferation, its basal-like expression profile and its actual origin from infiltrating ductal BC [32]. The list of human BC cell lines similar to MDA-MB-231 is long. While the majority of *in vitro* studies related to the function of E-cadherin in BC have been conducted with cell lines such as MDA-MB-231, these cell lines are inappropriate as models for ILC because they have not based their tumor evolution on E-cadherin loss. Authentic human ILC cell lines are rare and therefore studying E-cadherin function in *bona fide* ILC cells is just in its beginnings [14,33]. The following section describes human ILC cell lines and their molecular properties.

**Table 1:** **Human infiltrating lobular breast cancer cell models**

			**Protein expression**				**Mutational status**			**Molecular subtype**	
	**Tumor origin**	**Tissue**	**Ecad**	**ER**	**PR**	**ERBB2**	***CDH1***	***TP53***	***CTNNA1***		**References**
Cell lines											
MDA-MB-134	Unknown (ILC?)	A	Neg	Pos	Neg	Neg	mut^a^	wt/mut^d^	wt	lum	[25,30,34-40,45]
MDA-MB-330	ILC	P	Pos	Neg	Neg	Pos	wt	mut^e^	mut^i^	ErbB2-pos	[26,46]
MA-11	Mixed ITC/ILC	BM	Pos	Neg	Neg	Neg	NA	NA	NA	NA	[27]
SUM-44PE	Unknown (ILC?)	P	Neg	Pos	Pos	Neg	mut^b^	mut^f^	wt	lum	[28,33,36-38]
HCC-2185	ILC	P	NA	Neg	Neg	Pos	NA	NA	NA	lum	[24,29]
IPH-926	ILC	A	Neg	Neg	Neg	Neg	mut^c^	mut^g^	wt	lum	[30,33,41-43,70]
BCK-4	Unknown (muc-ILC?)	P	Neg	Pos	Pos	Neg	na	na	NA	NA	[31]
Xenograft tumors											
HBCX-7	ILC	PT	NA	Neg	Neg	Neg	NA	wt	NA	NA	[48]
HBCX-19	ILC	PT	NA	Neg	Neg	Neg	NA	mut^h^	NA	NA	[48]
HBCX-36	ILC	PT	NA	Pos	Pos	Pos	NA	NA	NA	NA	[49]
HCI-005	Mixed ILC/IDC	P	Pos	Pos	Pos	Pos	NA	NA	NA	NA	[47]
HCI-013	ILC	P	Neg	Pos	NA	NA	NA	NA	NA	NA	[36]

All information compiled from the literature; molecular subtype determined by microarray expression profiling in different studies. A, malignant ascites; BM, bone metastasis; Ecad, E-cadherin; ER, estrogen receptor; IDC, infiltrating ductal breast cancer; ILC, infiltrating lobular cancer; ITC, infiltrating tubular cancer; lum, luminal; muc-ILC, ILC with extracellular mucin; mut, mutated; NA, not assessed; neg, negative; P, malignant pleural effusion; pos, positive; PR, progesterone receptor; PT, primary breast tumor; wt, wild-type. ^a^c.688del145; p.L230EfsX4. ^b^c.1269delT; p.F423LfsX8. ^c^c.241ins4;p.V82fsX93. ^d^c.853G > A; p.E285K. Different mutational status in different studies; possibly a TP53-mutated subclone exists. ^e^c.659C > T; p.Y220C. ^f^c.82_84delinsCA; pE28fsX16. ^g^c.853G > A; p.E285K. ^h^Sequence unknown. ^i^c.1322C > G; p.S441X.

### *In vitro* models based on human infiltrating lobular breast cancer cell lines

The MDA-MB-134 cell line was initially reported to derive from infiltrating ductal BC [25]. Reis-Filho and colleagues reclassified this cell line as ILC (Table 1) [34]. MDA-MB-134 is E-cadherin-negative and ER-positive and belongs to the luminal molecular subtype [24]. MDA-MB-134 harbors a homozygous deletion of *CDH1* exon 6, which results in a frameshift and a premature stop codon [30,35]. Proliferation of MDA-MB-134 is moderately fast (doubling time of about 2 days) and depends on estrogenic stimulation [36,37]. MDA-MB-134 harbors a gain at chromosome 8p11-p12, an amplicon also common in primary ILCs [34]. MDA-MB-134 overexpresses FGFR1, which maps to chromosome 8p11-p12, and small interfering RNA-mediated silencing or inhibition of FGFR1 increases sensitivity to estrogen withdrawal or tamoxifen [36,38]. Accordingly, FGFR1 is thought to induce endocrine resistance in ILC. This is of relevance because endocrine control is the most important pharmacological treatment strategy for patients with ILC [16]. However, MDA-MB-134 cells also overexpress ZNF703, a newly identified oncogene involved in endocrine resistance. The *ZNF703* gene is located <1 Mb upstream of *FGFR1* and small interfering RNA-mediated silencing of *ZNF703* also decreases viability of MDA-MB-134 [39]. Using MDA-MB-134 as a model, recent studies proposed that tamoxifen has a partially agonistic activity in ILC. According to these studies, ILC proliferation is induced rather than inhibited by tamoxifen, an effect attributed to ZNF703 [36,40]. An MDA-MB-134 subclone with an activating mutation of the *KRAS* oncogene and altered response to FGFR1 inhibition has also been reported [38].

The SUM-44PE cell line is another accepted ILC model (Table 1) [36,37]. SUM-44PE is E-cadherin-negative and ER-positive and was derived from a malignant pleural effusion. The corresponding primary tumor, presumably an ILC, remained uncharacterized [28]. Compared with MDA-MB-134, SUM-44PE has a shorter doubling time (approximately 1 day), which may be due to amplification of cyclin D_1_ (*CCND1*), and is also responsive to steroid hormones. SUM-44PE harbors homozygous frameshift mutations in the *CDH1* and *TP53* tumor suppressor genes [35]. The SUM-44LCCTam subclone was established by chronic *in vitro* selection of SUM-44PE against tamoxifen. SUM-44-LCCTam cells overexpress ERRγ, an orphan nuclear receptor, which induces endocrine resistance [37]. Like MDA-MB-134, SUM-44PE cells overexpress FGFR1. Contrary to MDA-MB-134, silencing of FGFR1 only modestly increases sensitivity to estrogen withdrawal or tamoxifen [38].

The IPH-926 cell line was derived from malignant ascites of a metastatic ILC [30]. The corresponding primary tumor, a grade 1 ER-positive ILC, was diagnosed 16 years before establishment of the cell line (Figure 1B). The patient had undergone breast-conserving surgery and adjuvant tamoxifen therapy but experienced local and distant recurrences. The tumor recurrences had converted to an ER-negative status and histological grade 3, corresponding to a secondary pleomorphic phenotype [41]. Further treatment included conventional chemotherapies. The IPH-926 cell line was established from the endocrine-resistant and chemotherapy-resistant progressive disease [30]. *In vitro*, IPH-926 cells grow in loosely adherent grape-like clusters, but also form some single-file linear cords reminiscent of primary ILC (Figure 1B, arrow). IPH-926 harbors a unique homozygous *CDH1* frameshift mutation and lacks E-cadherin. Detection of the same *CDH1* mutation in archival tissue of the original ER-positive breast tumor verified the clonal origin of IPH-926 from ILC [30]. p120 relocates to the cell membrane upon reconstitution of E-cadherin in IPH-926 (Figure 1C) [33]. IPH-926 cells are ER/progesterone receptor (PR)/ERBB2 (triple)-negative, but retain a luminal subtype, as defined by microarray profiling [42]. IPH-926 has also retained a chemoresistant phenotype, which depends on an endogenous overexpression of the ABCB1/MDR1 xenobiotic transporter [43]. Cell proliferation of IPH-926 is slow (doubling time of 14 days) and independent from estrogenic stimulation. This seems related to an overexpression of *BCAR4*, a mediator of endocrine resistance [43,44]. Like MDA-MB-134 and SUM-44PE, IPH-926 harbors a gain at chromosome 8p12-p11. However, it lacks overexpression of FGFR1 and is not sensitive to FGFR1 inhibition [30]. In their *in vivo* clonal ancestry, IPH-926 cells acquired an additional *TP53* mutation [41]. The p53 mutant expressed in IPH-926, E285K, has temperature-sensitive loss of function characteristics. Interestingly, activation or inactivation of p53 has little impact on cell cycle distribution or apoptosis in these cells. Instead, restoration of p53 activity results in a metabolic suppression. Microarray analyses identified p53-regulated genes associated with this metabolic suppression, one of which is the AKT-inhibitor *PHLDA3* [41]. Notably, p53 E285K is also evident in a subclone of MDA-MB-134 [35,45] and has repeatedly been detected in therapy-refractory ILC [17].

Few other cell lines from ILC have ever been reported (Table 1) [26,27,29,31]. The MDA-MB-330 cell line expresses wild-type but dysfunctional E-cadherin due to a biallelic mutation in α-catenin (*CTNNA1*), which may represent an alternate mechanism to impair E-cadherin function [46]. The BCK-4 cell line was derived from an ILC with extracellular mucin, an exceptionally rare ILC variant [31].

The three most intensively investigated models (MDA-MB-134, SUM-44PE and IPH-926) have some features in common. These commonalities include a metastatic origin, mutation of *CDH1* and *TP53*, a luminal molecular subtype and amplification of chromosome 8p12-p11. All three cell lines lack *PIK3CA* hot-spot mutations common in primary ILC. As stated above, *TP53* mutations are rare in primary ILC, except for the pleomorphic variant. The accumulation of *TP53* mutations in the few available ILC cell lines may suggest a selection bias. Indeed, p53-deficient tumor cells are notorious for their superior *in vitro* growth. Establishment of a cell line from human nonmetastatic ILC with wild-type p53 and an activating *PIK3CA* mutation has not been achieved. Hence, human ILC cell lines have limitations regarding their representativeness for primary ILC, but have provided insight into mechanisms of endocrine resistance, chemoresistance and tumor progression.

### Infiltrating lobular breast cancer xenograft models

Engraftment of human tumor tissues into immunodeficient mice promises exact phenocopying of BC subtypes [47]. However, only few ILC xenografts have ever been described (Table 1) [36,47-49]. Tumor take rates are generally low for ER-positive BCs (approximately 2 to 4%) [48,49]. Cottu and colleagues, using Swiss nude mice as hosts, reported that tumor take was a modest 1/59 (1.7%) for ER-positive ILC [49]. The ER-positive ILC that did engraft was ERBB2-positive, which is uncharacteristic for ILC.

The low tumor take rate of ILC could occur for several reasons. First, correct macroscopic identification of ILC areas in human breast resection specimens is challenging. This is due to the often sparse cellularity of ILC. Hence, it is nearly impossible to control for the number of tumor cells transplanted. Second, the slow proliferation of ILC is probably not compatible with xenograft models. Development of a large tumor from a small tissue fragment may take several years and extend beyond the host’s lifespan.

Nonetheless, Sikora and colleagues established HCI-013 ILC xenograft tumors in nonobese diabetic/severe combined immunodeficient mice. Estrogen withdrawal decreased the time to tumor detection in this model [36]. Hence, HCI-013 recapitulates estrogen-dependent growth *in vivo*. Finally, ILC xenografts have also been generated by orthotopic or subcutaneous inoculation of human ILC cell lines. IPH-926 xenografts show histomorphological features reminiscent of human primary ILC [30]. BCK-4 xenografts switch from mucinous to lobular histology when supplemented with estrogens [31]. However, due to low tumor take rates, ILC xenograft models are currently of limited utility for ILC research.

### Sporadic infiltrating lobular breast cancer in animals

Before discussing GEM models, it is reasonable to ask whether ILC occurs as a sporadic tumor in animals. Sporadic BCs are well studied in dogs and cats, which, as pet animals, undergo surgical tumor resections. Current veterinary classification systems for canine, feline and rodent mammary tumors do not cover ILC as a naturally occurring entity [50,51]. However, Ressel and colleagues reviewed nearly 4,000 canine BCs and identified three cases of ILC [52]. Canine ILCs were E-cadherin-negative but were also ER/PR-negative, suggesting species-specific differences in hormonal growth control. E-cadherin-deficient LCIS has been reported in primates, but ILC is not known [53]. Hence, ILC is primarily a human disease and is very rare in domestic or free-ranging animals.

### E-cadherin knockout is lethal in conventional genetically engineered mouse models

GEM models have revolutionized cancer research [54]. There are three reasons for the success of GEM tumor models. First, mice are mammals. Second, mice share genetic similarities with humans. Third, the mouse germline can be easily manipulated to induce overexpression or knockout of target genes.

Early conventional GEM tumor models were based on the activation or inactivation of a single gene in the germline or large tissue compartments. This only crudely mimicked human tumorigenesis. Furthermore, embryonic lethality was a main drawback of the conventional GEM models. This is exemplified by knockout of *Cdh1* in the mouse germline. Homozygous loss of E-cadherin (*Cdh1*^−/−^) is lethal due to defective blastocyst formation [55]. Heterozygous mice (*Cdh1*^+/−^) develop normally and show no increased tumor incidence, suggesting that either E-cadherin haploinsufficiency does not induce tumors, E-cadherin loss is not tolerated and/or that the lifespan of the mouse is not sufficient to allow evolutionary loss of heterozygosity [55].

Although conventional *Cdh1* knockout was of little immediate value for elucidating the tumor suppressor function of E-cadherin, it inspired many decisive studies on the important roles of E-cadherin in murine embryonic stem cells and embryonic development [56]. Heterozygous mice (*Cdh*1^+/−^) have also been employed to establish a model resembling gastric signet ring cell carcinoma (a form of DGC) [57]. Exposure to carcinogenic *N*-methyl-*N*-nitrosourea via drinking water induced a 10-fold increase of E-cadherin-negative signet ring cell carcinomas in heterozygous mice (*Cdh1*^+/−^) compared with wild-type mice (*Cdh1*^+/+^) (Table 2) [57]. This study clearly implicated loss of E-cadherin as a second and collaborating hit in tumor formation and provided a compelling example of how genetic and environmental factors cooperate in the initiation of distinct tumors. The lack of an equivalent ILC model based on heterozygous (*Cdh1*^+/−^) mice may be for several reasons. One aspect is that carcinogens involved in gastric tumorigenesis are well defined, while environmental factors associated with BC are complex and cannot easily be adopted for laboratory animals.

**Table 2:** **Genetically engineered mouse models related to infiltrating lobular breast cancer**

**Promoter element**	**Recombinase/transgene**	**Targeted tissues**	**Conditional knockout**	**Tumors**	**Tumor spectrum (mammary gland or stomach)**	**References**
Mammary gland						
*MMTV*	*Cre*	MG^a^, SALG, SV	*Cdh1* ^*F/F*^	No	x	[59]
*K14*	*Cre*	MG^a^, SALG, EPD, ESO	*Cdh1* ^*F/F*^	No	x	[60]
*K14*	*Cre*	MG^a^, SALG, EPD, ESO	*Cdh1* ^*F/F*^	No	x	[61]
*WAP*	*Cre*	MG^a^	*Cdh1* ^*F/F*^	No	x	[62]
*WAP*	*Cre*	MG^a^	*Cdh1* ^*F/F*^	No	x	[63]
*K14*	*Cre*	MG^a^, SALG, EPD, ESO	*Cdh1* ^*F/F*^ *; Trp53* ^*F/F*^	Yes	mILC (54%), CS (27%), sAC (23%)	[61]
*WAP*	*Cre*	MG^a^	*Cdh1* ^*F/F*^ *; Trp53* ^*F/F*^	Yes	mILC (60%), sC/CS (67%), AC (2%)	[63]
Stomach						
x	*Cdh1* ^*+/−*^	None	x	No	x	[57]
x	*Cdh1* ^*+/−*^ (+MNU)	None	x	Yes	tubAd (75%), SRCC (45%)	[57]
*ATP4B*	*Cre*	GPC	*Cdh1* ^*F/F*^	No	(noninvasive signet ring cell clusters)	[64]
*ATP4B*	*Cre*	GPC	*Cdh1* ^*F/F*^ *; Trp53* ^*F/F*^	Yes	DGC	[65]
*PDX1*	*Cre*	GMC, DMC, PIC	*Cdh1* ^*F/+*^ *; Smad4* ^*F/F*^ *; Trp53* ^*F/F*^	Yes	DGC (85%, Ecad-negative), DDAC (26% Ecad-positive), SCC (24%)	[66]

All information compiled from the literature. AC, adenocarcinoma; CS, carcinosarcoma; DGC, diffuse gastric cancer; DDAC, duodenal adenocarcinoma; DMC, duodenal mucosal cells; Ecad, E-cadherin; EPD, epidermis; ESO, esophageal mucosa; GMC, gastric mucosal cells; GPC, gastric parietal cells; MG, mammary gland; mILC, murine infiltrating lobular carcinoma; MNU, carcinogenic *N*-methyl-*N*-nitrosourea given with the drinking water; sAC, solid adenocarcinoma; sC, solid carcinoma; PIC, pancreatic islet cells; SCC, squamous cell carcinoma; SALG, salivary gland; SRCC, signet ring cell carcinoma; SV, seminal vesicle; tubAd, tubular adenoma. ^a^Luminal and myoepithelial cells in virgin and pregnant female mice.

### E-cadherin knockout is not tumorigenic in conditional genetically engineered mouse models

To study all properties of BC pathology, GEM models are needed that mimic not only human tumor phenotypes but also their initiation from individual cells in adult tissues. Conditional GEM tumor models based on site-specific recombination systems, such as Cre/loxP from bacteriophage P1, allow for somatic and stochastic mutation of target genes in defined tissues of a wild-type background [58]. A number of different approaches have been employed, using different promoter elements driving *Cre* recombinase expression to cell type-specific *Cdh1* ablation in the mouse mammary gland and gastrointestinal tract [59-66] (Table 2).

The common denominator of these studies is that mice fail to develop BC upon conditional knockout of E-cadherin using either *K14*, *WAP* or *MMTV* as Cre recombinase drivers. The underlying reason for this phenomenon is the fact that loss of E-cadherin is not tolerated in the luminal epithelial compartment of the mouse mammary gland. Depending on the promoter driving *Cre*, E-cadherin ablation will result in massive apoptosis (*MMTV*) or nearly undetectable clearance of E-cadherin-deficient luminal cells (*K14*) [59,61-63]. Intriguingly, human E-cadherin-negative LCIS can subsist for years without regression or progression. Based on data from mouse models, this implies either that a primary oncogenic hit allowing E-cadherin loss is already present in human ILC or, in contrast to the mouse, human luminal mammary cells can cope with E-cadherin loss. The latter option may be explained by redundancy mechanisms. *Cdh1* knockout in the basal stratified and follicular epidermal cells of the skin induces a compensatory upregulation of P-cadherin (*Cdh3*) that rescues epithelial integrity in the basal layer of the epidermis, but not in the hair follicle [60]. Since luminal epithelial cells exclusively express E-cadherin and myoepithelial cells express P-cadherin, this seems an unlikely scenario for the mammary gland. *Cdh1* ablation in the gastric mucosa also did not result in gastric cancer, although noninvasive E-cadherin-negative cell aggregates occurred [64]. Together, conditional GEM models imply that additional oncogenic hits are compulsory in the mammary gland before *Cdh1* inactivation can be tolerated and unleash its full tumorigenic potential.

### Compound genetically engineered mouse models provide insight into infiltrating lobular breast cancer biology

Given that somatic inactivation of E-cadherin as a primary hit is not tolerated in the breast, compound conditional GEM models have been developed based on concomitant inactivation of p53 and E-cadherin. Somatic inactivation of p53 also allowed study of the E-cadherin function in tumor progression, because mammary-specific inactivation of p53 alone resulted in nonmetastatic, locally expansive tumors [61,63]. In contrast, dual conditional knockout of E-cadherin and p53 using either *K14cre* or *WAPcre* synergized with p53 loss and induced a dramatic shift from expansive to infiltrating growth. Homozygous loss of E-cadherin led to the formation of carcinomas that phenocopied human ILC. While from a cytopathological perspective most of these tumors showed extensive nuclear pleomorphism, due to their striking similarity to human ILC invasion patterns they were designated as mouse ILC (Figure 1D). Based on mRNA profiling and expression of cytokeratin 8, mouse ILC is luminal in character but does not express ER/PR during the later stages of tumor progression. Since conditional mouse BC models in general show absence of ER and PR expression, this probably reflects species-specific physiological differences. Of importance, however, is the finding that mouse ILC shows a dissemination spectrum similar to human ILC, with specific metastasis to the gastrointestinal tract and peritoneum and common sites such as the lung and bone marrow [61,63]. Chemotherapy prolongs survival but does not eradicate metastases in this model [67]. While antihormone therapy can keep human metastatic ILC under control, endocrine-resistant ILC is notorious for poor response to chemotherapy [68]. Mouse ILC models are therefore better categorized as models for endocrine-resistant and chemorefractory metastatic ILC.

Availability of the mouse ILC models has provided new opportunities to study ILC cell biology. Reconstitution of E-cadherin in p53-deficient mouse ILC cell lines abolished their ability to proliferate under anchorage-independent conditions, showing causality for the loss of E-cadherin in this process [61]. Follow-up studies showed that, unlike β-catenin, p120 is not proteosomically degraded in ILC but instead resides in the cytosol and nucleus upon E-cadherin loss. Cytosolic p120 (a distinguishing feature of human ILC) controls Rho/Rock-dependent anoikis resistance of ILC by binding and inhibition of the Rho antagonist Mrip [14]. While it is still unclear how p120 triggers anchorage-independent survival distal from Rho and Rock, future answers may come from the ability of p120 to inhibit transcriptional repression by Kaiso [69]. ILC is characterized by a decrease in nuclear Kaiso and relief from p120-dependent Kaiso repression [70]. Our unpublished data have also identified noncanonical Wnt11 as a Kaiso target driving autocrine Rho-dependent anoikis resistance (van de Ven RAH, unpublished data), suggesting that p120 is a multifaceted oncoprotein in ILC. These findings furthermore denote options for future intervention because ILC progression depends on Rho/Rock signaling, a pathway that is susceptible to pharmacological inhibition.

Interestingly, indirect mammary-specific loss of E-cadherin function through ablation of p120 did not induce murine ILC. In this context, p120 functioned as a tumor suppressor, and its loss in *WAPcre*;*Ctnnd1*^F/F^;*Trp53*^F/F^ female mice led to dismantling of the E-cadherin-dependent cell–cell adhesion and formation of metastatic tumors that resembled metaplastic triple-negative BC [71]. These studies also showed that inactivation of the AJ partially controls anchorage independence through hypersensitization of endogenous growth factor receptors. This phenomenon appeared independent of the phenotypic outcome, but was dependent on the absence of cadherin-based AJs [71]. These data may provide an explanation for the prevalence of oncogenic events that lead to activation of PI3K/AKT-dependent cues in ILC. Moreover, they suggest that ILC patients may be eligible for clinical interventions using therapies that target growth factor receptor signaling, especially in the absence of activating mutations or amplifications.

Comparable compound conditional GEM models have also been established for gastric cancer. Inactivation of E-cadherin and p53 in *ATP4Bcre*;*Cdh1*^*F/F*^;*Trp53*^F/F^ mice resulted in the progression of E-cadherin-negative gastric mucosal cell aggregates to invasive and metastatic tumors resembling human DGC [65] (Table 2). In another compound model, E-cadherin-deficient DGCs developed in *PDX1*cre;*Cdh1*^F/+^;*Trp53*^F/F^*Smad4*^F/F^ mice, suggesting a selection pressure for spontaneous inactivation of a remaining wild-type *Cdh1* allele during gastric tumorigenesis [66].

Taken together, several conditional compound GEM models involving somatic knockout of *Cdh1* recapitulate ILC-like or DGC-like tumors (Table 2). Follow-up studies showed that *Cdh1* inactivation releases p120 from AJs to the cytosol and nucleus, where it controls tumor progression through distinct and druggable signaling pathways. Blocking these pathways may therefore be a rational strategy for the design of targeted therapies to better treat metastatic ILC.

## Conclusions

ILC is the most common special BC subtype. With mutational or epigenetic inactivation of E-cadherin being confined almost exclusively to ILC and LCIS, this tumor entity stands out from all other kinds of BCs. The molecular basis of ILC is clearly linked to loss of E-cadherin, as evidenced by hereditary cases associated with *CDH1* germline mutation and conditional knockout mouse models.

What is the big picture we obtain from ILC models? ILC is difficult to study on a functional level. Human ILC cell lines are rare. All of them are of metastatic origin and harbor mutant p53. Their biology reflects end-stage ILC progression. Primary ILCs show almost no tumor take in xenograft models. Currently established GEM models phenocopy ILC but lack ER expression. Nonetheless, ILC models have advanced our understanding of this disease tremendously. Several new candidate genes and signaling pathways have come to the fore of ILC biology. These include FGFR1, ZNF703, ERRγ, BCAR4 and ABCB1/MDR1 as mediators of therapy resistance and mutant p53 as the gatekeeper towards pleomorphic ILC. Furthermore, p120 has been confirmed to mediate multiple oncogenic signals through Rho-Rock signaling upon inactivation of E-cadherin.

What are the most important questions that remain to be explored? It is still unclear how loss of E-cadherin impacts on gene expression patterns and differentiation of developing ILC. Microarray analyses following E-cadherin reconstitution in ILC cells might be instructive. Our own profiling of IPH-926 and MDA-MB-134 reconstituted with E-cadherin has so far revealed little or no reorganization of the transcriptome (Karch I, unpublished observation). This makes the mystery surrounding E-cadherin even more intriguing.

Regarding mechanisms of endocrine resistance, collaboration between basic scientist, histopathologists and clinicians is warranted [72]. Current clinical trials addressing pretreatment/post-treatment biomarker changes in BC patients receiving neoadjuvant endocrine therapy establish an invaluable resource of tumor tissues informative for endocrine responsiveness. Future translational research studies will take advantage of this resource to determine the predictability of clinical endocrine resistance using surrogate markers from ILC models, such as FGFR1, ZNF703 and BCAR4.

In the field of GEM models, conditional inactivation of *Cdh1* combined with activation of latent mutant *Pik3ca*^H1047R^ promises tumors with unique, perhaps ILC-like properties. The most important challenge, however, is to understand and target the mechanisms that counteract the proapoptotic signals upon loss of E-cadherin in LCIS and ILC. These mechanisms might be heterogeneous and could predestinate for metachronous cancer development in the one LCIS patient but not in another. The role of a recently identified ILC-specific single nucleotide polymorphism on chromosome 7q34 will also be of interest in this context [73]. Definition of prognostically favorable and unfavorable LCIS would be a major achievement, since LCIS is common and the risk of progression to invasive ILC is as yet entirely unpredictable on an individual patient basis.

## Abbreviations

AJ: Adherens junction
BC: Breast cancer
DGC: Diffuse gastric cancer
ER: Estrogen receptor
GEM: Genetically engineered mouse
ILC: Infiltrating lobular breast cancer
LCIS: Lobular carcinoma *in situ*
p120: p120-catenin
PR: Progesterone receptor
